# Loss-of-Function Variants in *EFEMP1* Cause a Recognizable Connective Tissue Disorder Characterized by Cutis Laxa and Multiple Herniations

**DOI:** 10.3390/genes12040510

**Published:** 2021-03-31

**Authors:** Maxim Verlee, Aude Beyens, Alper Gezdirici, Elif Yilmaz Gulec, Lore Pottie, Silke De Feyter, Michiel Vanhooydonck, Piyanoot Tapaneeyaphan, Sofie Symoens, Bert Callewaert

**Affiliations:** 1 Center for Medical Genetics Ghent, Ghent University Hospital, 9000 Ghent, Belgium; maxim.verlee@ugent.be (M.V.); aude.beyens@ugent.be (A.B.); Lore.Pottie@UGent.be (L.P.); Silke.DeFeyter@uzgent.be (S.D.F.); Michiel.Vanhooydonck@UGent.be (M.V.); Piyanoot.Tapaneeyaphan@UGent.be (P.T.); Sofie.Symoens@UGent.be (S.S.); 2 Department of Biomolecular Medicine, Ghent University, 9000 Ghent, Belgium; 3 Department of Dermatology, Ghent University Hospital, 9000 Ghent, Belgium; 4 Department of Medical Genetics, Basaksehir Cam and Sakura City Hospital, 34480 Istanbul, Turkey; dralpergezdirici@gmail.com; 5 Department of Medical Genetics, Kanuni Sultan Suleyman Training and Research Hospital, Health Sciences University, 34303 Istanbul, Turkey; mdegulec@gmail.com

**Keywords:** *FBLN3*, *EFEMP1*, fibulin-3, cutis laxa, elastic fiber, extracellular matrix, inguinal hernia, diaphragmatic hernia

## Abstract

Hereditary disorders of connective tissue (HDCT) compromise a heterogeneous group of diseases caused by pathogenic variants in genes encoding different components of the extracellular matrix and characterized by pleiotropic manifestations, mainly affecting the cutaneous, cardiovascular, and musculoskeletal systems. We report the case of a 9-year-old boy with a discernible connective tissue disorder characterized by cutis laxa (CL) and multiple herniations and caused by biallelic loss-of-function variants in *EFEMP1*. Hence, we identified *EFEMP1* as a novel disease-causing gene in the CL spectrum, differentiating it from other HDCT.

## 1. Introduction

Hereditary disorders of connective tissue (HDCT) are a heterogeneous group of diseases, mainly affecting the cutaneous, ocular, cardiovascular, pulmonary, and musculoskeletal systems. HDCT are caused by pathogenic variants in genes encoding structural and regulatory components of the extracellular matrix (ECM) [1].

Fibulins belong to a protein family implicated in both elastic fiber (EF) assembly and function. Loss-of-function variants in *FBLN4* and *FBLN5* both cause severe recessive cutis laxa (CL), characterized by loose redundant skin folds and variable systemic involvement, including prominent emphysema [2,3]. Fibulin-3, encoded by *EFEMP1*, is a secreted extracellular matrix glycoprotein, which is abundantly expressed in skin fibroblasts, retina, fascia, and vasculature [4]. It is believed to function as an important modulator of ECM biology via its interaction with various ECM molecules, including tropoelastin and (tissue inhibitors of) matrix metalloproteinases [5]. A recurrent gain-of-function variant (p.(Arg345Trp)) in *EFEMP1* has previously been associated with Doyne honeycomb retinal dystrophy [6]. However, biallelic pathogenic variants in *EFEMP1* were recently reported in two probands with a pronounced connective tissue phenotype, characterized by multiple herniations and joint hypermobility [7,8].

In this study, we present a 9-year-old Turkish boy with a novel, discernible connective tissue disorder characterized by cutis laxa and multiple herniations, associated with biallelic loss-of-function variants in *EFEMP1*.

## 2. Materials and Methods

### 2.1. Consent

The legal guardian of the patient in this manuscript gave written informed consent to publication of the case details. Specific informed consent was obtained for the publication of clinical pictures. This study was conducted in accordance with the 1984 Declaration of Helsinki and its subsequent revisions.

### 2.2. Molecular Analysis

Exome sequencing (ES) of the proband was performed on genomic DNA extracted from peripheral leukocytes. Target enrichment of the gDNA was obtained by the SureSelectXT Low Input Human All Exon V7 (Agilent Technologies, Santa Clara, CA, USA). Sequencing was performed on a NovaSeq 6000 platform (Illumina, San Diego, CA, USA) with a minimal expected coverage depth of 20×. All obtained variants were analyzed using the Seqplorer software, an in-house developed pipeline, which integrates population frequencies (GnomAD) [9] and in silico splice predictions (e.g., Mutationtaster, PolyPhen, SIFT (sorting tolerant from intolerant), CADD (Combined Annotation Dependent Depletion), Revel (Rare Exome Variant Ensemble Learner), Ada (Adaptive boosting), and RF (Random Forest scores) [10,11,12,13,14,15]. Variants with a minor allele frequency > 0.01 in the GnomAD database were filtered out and the remaining variants were selected according to their functional consequences, whereas only splice-site and stop-gain variants, insertions, deletions, and missense variants were retained. Candidate variants were classified according to the refined American College of Medical Genetics and Genomics (ACMG) guidelines [16,17] and confirmed by Sanger Sequencing on an ABI 3730 platform (Applied Biosystems, Waltham, MA, USA) in the proband and both parents. Obtained sequence profiles were compared with the *EFEMP1* reference sequence (Refseq NM_001039348.3).

All variants were reported according to the nomenclature of the Human Genome Variation Society (HGVS, http://www.hgvs.org/VARONEM, accessed on 28 February 2021) [18].

## 3. Case Presentation

**Figure 1:**
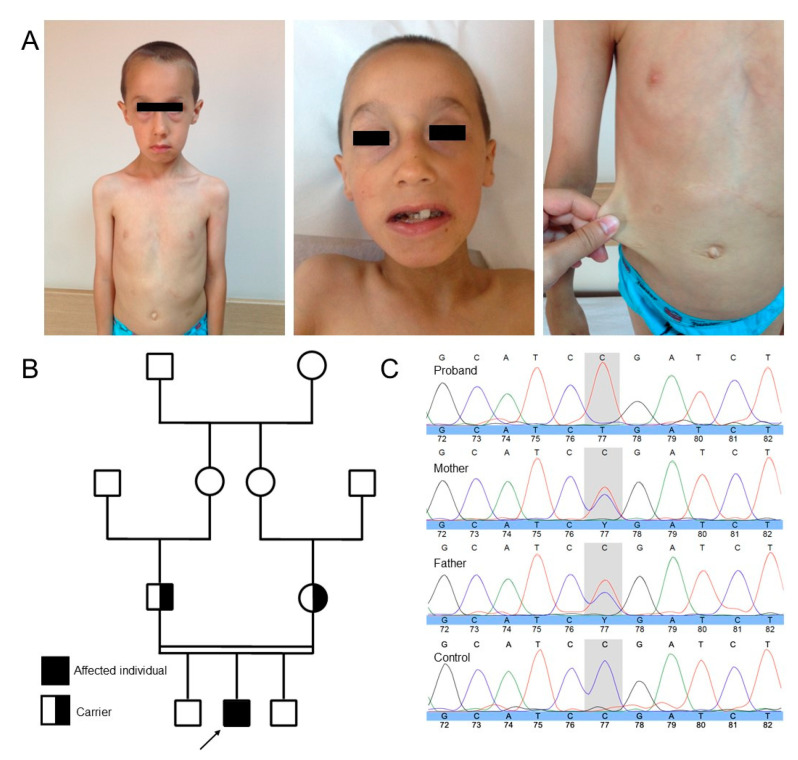
(**A**) Clinical photographs taken at age 7. Note the long face, high anterior hairline, telecanthus, blepharochalasis, sagging lower eyelids and cheeks, long philtrum, full lips, downturned mouth corners, retrognathia, dental caries and malocclusion, and cutis laxa. (**B**) Pedigree of the reported consanguineous family of Turkish descent. The proband is indicated by the black arrow. (**C**) Sanger sequencing confirms the presence of a homozygous *EFEMP1* nonsense variant (c.1201C > T, p.(Arg401*)) in the proband and identifies both parents as heterozygous carriers.

The proband is a boy with suspected HDCT. He was born at term after an uncomplicated pregnancy to consanguineous parents of Turkish origin. At birth, he presented with cutis laxa and a congenital diaphragmatic and right inguinal hernia, which were surgically corrected at the ages of 8 months and 1.5 years, respectively. During clinical examination upon referral aged 7, he showed failure to thrive (length: 112 cm (−0.67 SD); weight: 15.9 kg (−2.5 SD)), despite reportedly normal intake. His head circumference was normal (50 cm (−1.28 SD)). He presented with discernable craniofacial characteristics, including a long face, a high anterior hairline, telecanthus, blepharochalasis, sagging lower eyelids and cheeks, a long philtrum, full lips, downturned mouth corners, retrognathia, dental caries, malocclusion, and cutis laxa (Figure 1A). He further presented with muscle hypotonia and generalized joint hypermobility. His skin was markedly redundant, thin, translucent, and bruised easily. Scars healed normally. Routine laboratory tests including peripheral blood cell analysis, liver and kidney function tests, and coagulation were normal. Although his initial neuromotor development was normal, he showed mild intellectual disability (ID). Hearing, vision, the cardiovascular system, and the urinary tract were assessed as normal. Aged 9, he developed a left inguinal hernia. Exome sequencing identified a homozygous nonsense variant (c.1201C > T, p.(Arg401*)) in *EFEMP1* (Figure 1C). This variant is absent in the Gnomad exomes and genomes databases, is classified as “pathogenic” by all used in silico prediction programs, and segregates with disease and carrier status in the reported family.

## 4. Discussion

We present a 9-year-old Turkish boy with a discernible connective tissue disorder consisting of cutis laxa and multiple herniations with homozygous loss-of-function mutations in *EFEMP1* (OMIM#601548), which encodes fibulin-3. Several lines of evidence support the causality of the identified loss-of-function variant for the observed phenotype in the reported individual. 

Firstly, fibulin-3 belongs to a family of 8 ECM glycoproteins associated with basement membranes and elastic fibers and is abundantly expressed in skin fibroblasts and fascia [4]. Fibulin-3 is structurally most related to fibulin-4 and -5, both critical molecules for EF assembly. Pathogenic variants in *FBLN4* (*EFEMP2*) and *FBLN5* cause autosomal recessive cutis laxa (ARCL) type 1b and 1a, which are associated with severe vasculopathy and developmental pulmonary emphysema, respectively. Of note, the latter condition is also associated with diverticula of the gastrointestinal and genitourinary system, as well as congenital diaphragmatic hernia. ARCL1c, a third disorder within the ARCL type 1 spectrum, shows considerable overlap with ARCL1a and is caused by pathogenic variants in *LTBP4*, which is an interaction partner of both *FBLN4* and *FBLN5* [2,3,19]. 

Secondly, *Efemp1*-knockout mice demonstrate herniations, loose skin, small body size or mass, atrophy of muscle and fat tissue, and dermal EF fragmentation. Interestingly, the resulting phenotype is highly dependent on the genetic background of the mice, as herniations are predominantly manifested in *Efemp1*^−/−^ mice on the C57BL/6 background, but not the BALB/c background [20]. 

Thirdly, biallelic loss-of-function variants in *EFEMP1* have previously been reported in 3 individuals of 2 distinctive families with multiple herniations and joint hypermobility, supporting loss-of-function as the molecular mechanism of a novel heritable connective tissue disease (Table 1). Our patient shows significant clinical overlap with the previously reported patients, with hernias of the integument and diaphragm and the presence of discernible facial features (with a long face and downslanted palpebral fissures) [7,8,21]. In contrast to the previously published individuals, our patient did not present with scoliosis, pectus deformity, or myopia. He further exhibited mild ID, which usually manifests in patients with ARCL type 2 and 3, but has not been associated with ARCL type 1 [19,22,23]. Exome analysis did not identify other pathogenic variants that could explain the ID, however this does not exclude genetic and non-genetic causes of ID. Our proband had a normal cardiovascular and pulmonary work-up, while multiple pulmonary bullae developed during puberty in one individual with biallelic truncating variants in *EFEMP1* [8]. Although several mild cutaneous features were present in 3 patients, our proband was the only case exhibiting cutis laxa and easy bruising with infantile onset [7,8,21]. Of note, pulmonary emphysema was only reported in one patient [8]. Overall, this report adds evidence that biallelic loss-of-function variants in *EFEMP1* are associated with a specific connective tissue disorders characterized by multiple hernias and skeletal and pulmonary manifestations. Although clinically variable, this disorder should be classified within the group of ARCL type 1 [7,8,19,21]. 

In conclusion, biallelic loss-of-function variants in *EFEMP1* cause a novel HDCT characterized by cutis laxa and recurrent herniations. As these patients additionally present with mild cutaneous and pulmonary manifestations, we propose this HDCT to be a classified as a subtype of ARCL type 1. This observation confirms that *EFEMP1* has a pivotal role in abdominal wall integrity.

## Figures and Tables

**Table 1:** Summary of molecular and clinical characteristics of individuals with *EFEMP1* pathogenic variants and comparison with ARCL type 1.

	*EFEMP1*-Related CL				ARCL1a	ARCL1b	ARCL1c
	This study	Bizzari et al. [7]		Driver et al. [8]	Beyens et al. [24]		
		Mégarbané et al. [21]					
**Clinical characteristics**							
Craniofacial dysmorphism	+ ^a^	+ ^a^	+ ^a^	+ ^a^	+ ^b^	+ ^c^	+ ^d^
Dental crowding	−	+	+	−	−	−	−
Cutis laxa	+	−	−	−	+	+	+
Thin translucent skin	+	−	−	+	−	−	+
Diaphragmatic hernia	+	−	+	+	+	+	+
Inguinal hernia	+	+	+	+	+	+	+
Hypermobile joints	+	+	+	+	+	+	+
Muscle hypotonia	+	+	+	+	−	−	+
Scoliosis	−	−	+	+	−	+	−
Pectus deformities	−	+	+	+	−	+	+
Tall Stature	−	+	+	+	−	−	−
Aortopathy	−	−	−	−	+	+	+
Emphysema	−	−	−	+	+	−	+
Gastrointestinal abnormalities	−	+	+	−	−	−	+
Bladder diverticula	−	+	+	+	+	−	+
**Molecular characteristics**	*EFEMP1*	*EFEMP1*	*EFEMP1*	*EFEMP1*	*FBLN5*	*FBLN4*	*LTBP4*
cDNA level	c.1201C > T	c.163T > C	c.163T > C	c.320_324delTG GCA c. 615T > A			
Protein level	p.(Arg401*)	p.(Cys55Arg)	p.(Cys55Arg)	p. (Met107fs) p.(Tyr205*)			
Zygosity	Homozygous	Homozygous	Homozygous	Heterozygous			
Other		*VCPKMT* and *MYO3* variants					

Note: ARCL, autosomal recesssive cutis laxa; +: characteristic present; −: not present; ^a^: long face, telecanthus, downslanted palpebral fissures; ^b^: high anterioir hairline, hypertelorism; ^c^: high anterior hairline, large ears, broad nose, sagging cheecks; ^d^: high anterior hairline, large ears, long philtrum; Grey background color indicates that the data refer to what is generally observed in ARCL1a, ARCL1b, or ARCL1c. Based on these data and the following papers: Bizzari et al. (2020) [7], Megarbane et al. (2012) [21], Driver et al. (2020) [8], and Beyens et al. (2020) [24].

## Data Availability

Data available in a publicly accessible repository. The data presented in this study are openly available in Clinvar, and the accession number is ‘SCV001548232.1’.
